# Hepatic 18F-FDG Uptake Measurements on PET/MR: Impact of Volume of Interest Location on Repeatability

**DOI:** 10.1155/2017/8639731

**Published:** 2017-05-30

**Authors:** Liran Domachevsky, Hanna Bernstine, Meital Nidam, Dan Stein, Natalia Goldberg, Dorit Stern, Ifat Abadi-Korek, David Groshar

**Affiliations:** ^1^Department of Nuclear Medicine, Assuta Medical Centers, Tel Aviv, Israel; ^2^Sackler Faculty of Medicine, Tel Aviv University, Tel Aviv, Israel

## Abstract

**Background:**

To investigate same day 18F-FDG (Fluorodeoxyglucose) PET (Positron Emission Tomography)/MR (Magnetic Resonance) test-retest repeatability of Standardized Uptake Value measurements normalized for body weight (SUV) and lean body mass (SUL) in different locations in the liver.

**Methods:**

This prospective study was IRB approved with written informed consent obtained. 35 patients (20 women and 15 men, 61 ± 11.2 years) that performed a whole-body 18F-FDG PET/MR followed by liver-dedicated contrast-enhanced 18F-FDG PET/MR were included. SUV/L max, mean, and peak were measured inferior to, superior to, and at the right portal vein and in the left lobe of the liver. The coefficient of variation (CV) and intraclass correlation coefficient (ICC) were calculated and Bland-Altman plots were obtained.

**Results:**

The variability for SUV/L's measurements was lowest inferior to the portal vein (<9.2%) followed by measurements performed at the level of the portal vein (<14.6%).

**Conclusion:**

The area inferior to the portal vein is the most reliable location for hepatic 18F-FDG uptake measurements on PET/MR.

## 1. Introduction

Tumoral FDG (Fluorodeoxyglucose) uptake reflects the metabolic activity of the tumor and is mainly used to evaluate tumor aggressiveness on baseline studies and to assess the response to treatment and prognosis based on interval changes of FDG uptake during and after treatment.

FDG uptake can be assessed either semiquantitatively or qualitatively. Semiquantitative assessment uses the Standardized Uptake Value (SUV) variable that can be used as a stand-alone variable or as a tumor to background ratio. Qualitative assessment is based on visual comparison of tumor uptake with tissues having different levels of FDG uptake. With either method of FDG uptake evaluation, the assumption is that background FDG uptake is reliable which is important for sequential studies of a single patient and between different patient groups.

It is therefore crucial to know FDG uptake variability of normal tissues in order to recognize changes that reside within the variability range and to rule out systemic errors that might occur whenever outside the range differences in variability are found.

In clinical practice, mainly for lymphoma, blood pool (e.g., mediastinum) and liver SUV measurements are frequently used in PET (Positron Emission Tomography)/CT (Computed Tomography) as background since these tissues have adequate test-retest repeatability [1].

In addition, given their different mean SUV measurements, a graded visual scale has been developed with two reference points (i.e., mediastinum and liver) instead of one, better reflecting the continuous nature of FDG uptake resulting in better stratification of the response to treatment [2].

As of now, test-retest repeatability of FDG uptake in the liver has been evaluated only with PET/CT [3]. Several studies used one area to measure FDG uptake while others used different areas within the liver. To the best of our knowledge, at present, no study has investigated which area of the liver has the most reliable SUV measurements on PET/MR (Magnetic Resonance).

The purpose of the current study is therefore to evaluate the test-retest repeatability of different SUV measurements normalized for body weight (SUV) and lean body mass (SUL) in different locations in the liver using PET/MR.

## 2. Methods

### 2.1. Subjects

This prospective study has been approved by the institutional review board. All subjects signed an informed consent form. Between September 2015 and March 2017, consecutive patients who performed whole-body nonenhanced 18F-FDG PET/MR followed by contrast-enhanced 18F-FDG PET/MR centered at the liver were enrolled. All patients performed 18F-FDG PET/CT prior to the 18F-FDG PET/MR.

### 2.2. PET/MR Protocol

18F-FDG PET/MR was performed from skull base to mid-thigh on the Biograph mMR (Siemens AG, healthcare sector, Erlangen, Germany) simultaneous PET/MR system. 18F-FDG injection dose was 5.18 MBq/kg.

Patients were positioned in a supine position and multistep/multibed scanning was performed in caudocranial direction with four bed positions. We used a 24-channel spine RF coil integrated within the MR bed and 3 surface body coils (6 channel each) to cover the thorax, abdomen, and pelvis. For the neck we used a 16-channel RF head/neck coil.

PET data was acquired in the list mode with the following reconstruction parameters: high definition PET +ordered subset expectation maximization (OSEM) iterative algorithm, three iterations and 21 subsets, and Gaussian filter: FWHM 4 mm; relative scattered correction.

For the nonenhanced scan each bed position was started with coronal Dixon-based sequences for MR attenuation correction (MRAC) (breath holding) (19 sec). This was followed by axial T2 HASTE (free breathing) (36 sec), coronal T2 HASTE with fat suppression (FS) (Inversion recovery- (IR-) based) (44 sec), and axial T1 VIBE Dixon (breath holding) (20 sec). PET data was acquired simultaneously with acquisition time of 5 minutes for each bed position.

These sequences were followed immediately by a liver-dedicated contrast-enhanced scan using Gadoteric acid (Dotarem®, Guerbet, France) (0.2 ml/kg, 0.1 mmol/kg at 1-2 ml/s, 20 ml saline flush) centered at the liver with the following parameters: Coronal Dixon-based sequences for MR attenuation correction (MRAC) (breath holding) (19 sec); nonenhanced Axial VIBE FS (breath holding) (18 sec) followed by three contrast-enhanced Axial VIBE FS (breath holding) each lasting 18 sec with 20 sec gap between scans. This was followed by a coronal 2D FLASH FS (breath holding) (18 sec) and late contrast-enhanced Axial VIBE FS (breath holding) (18 sec). PET data was again acquired simultaneously with acquisition time of 5 minutes.

**Figure 1:**
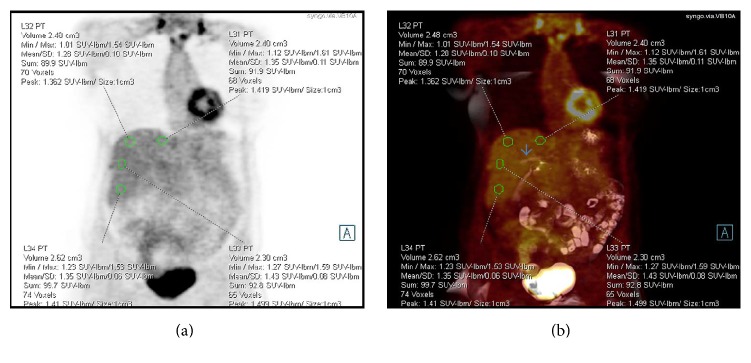
(a) Coronal PET attenuation correction image with spheres located inferior to, superior to, and at the level of the portal vein as well as in the left lobe of the liver. (b) Coronal fused T2-weighted HASTE FS PET/MR image demonstrating the spheres related to the portal vein (arrow).

SUV and SUL (mean, max, and peak) were measured using a sphere volume of interest (VOI) ranging between 2 and 3 ml (Figure 1). SUV/L max is a single-pixel value of the maximal SUV/L within the sphere, whereas SUV/L peak is the mean SUV/L within a predetermined volume of interest (VOI) of 1 ml around the voxel with the highest SUV/L in the sphere [4]. SUV/L mean is the average SUV/L value within the sphere.

Normalization for BW (body weight) was performed using the patient weight in kg, measured before 18F-FDG injection, and for LBM (lean body mass) using the following formula:

(1) LBM female=1.07×BW kg−148BW kgbody  height cm2,LBM male=1.1×BW kg−120BW kgbody  height cm2.

### 2.3. Image Analysis

We used dedicated software for SUV/L calculations (Syngo.via; Siemens AG, healthcare sector, Erlangen, Germany). A sphere VOI was drawn in four areas in the liver parenchyma: superior to, inferior to, and at the level of the portal vein and in the left lobe of the liver. The sphere was located on PET attenuation correction images of the whole-body PET/MR scan and on the PET attenuation correction images of the liver-dedicated scan after verifying the corresponding exact location and the lack of abnormalities in this area on all MR sequences (Figure 1). All measurements were conducted by a dual board-certified in radiology and nuclear medicine physician (L. D., with 3 years of experience).

### 2.4. Statistics

MedCalc (16.2.0) was used for all statistical analyses. Mean differences of the various SUVs/Ls between test and retest were calculated.

The coefficient of variation (CV) was calculated using the following formula:

(2) $$\begin{array}{c} \text{CV }\left(\;\%\;\right)=100\times \frac{SD}{mean}, \end{array}$$

where SD = √∑(*X*_1_ − *X*_2_)^2^/2*N* (within-patient variation), *X*_1_ and *X*_2_ represent test and retest measurements, and *N* denotes the number of patients.

The intraclass correlation coefficient (ICC) was used to estimate the absolute agreement among measurements to compensate for systematic differences. ICC was interpreted as follows: 0–0.2 indicated poor agreement; 0.21–0.4 indicates fair agreement; 0.41–0.6 indicates moderate agreement; 0.61–0.80 indicates good agreement; and > 0.80 indicates very good agreement. Bland-Altman plots were obtained to assess the metrics differences between test and retest.

## 3. Results

35 patients (20 women and 15 men, 61 ± 11.2 years) with cancer (Gastrointestinal: 16, genitourinary: 6, breast: 9, lymphoma: 3, and melanoma: 1) without conspicuous liver metastases or steatosis on CT and on the MR part of the study were enrolled. The 18F-FDG uptake period was 83 ± 15 minutes.

**Figure 2:**
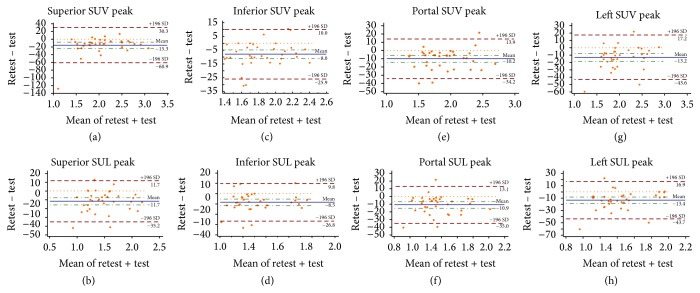
Bland-Altman plots of difference versus mean of SUV peak between retest and test in the superior (a, b), inferior (c, d), portal (e, f), and left (g, h) areas of the liver normalized to BW (a, c, e, g) and LBM (b, d, f, h). SUV: Standardized Uptake Value measurements normalized for body weight. SUL: Standardized Uptake Value measurements normalized for lean body mass.

Test-retest mean differences for the various SUV/L in the different regions of the liver are presented on Bland-Altman plots (Figure 2) and are shown in Table 1.

CV values of the various SUVs/Ls were always lower when measured inferior to the portal vein followed by measurements performed at the level of the portal vein. The highest CV's is seen superior to the portal vein and at the level of the left lobe of the liver (Table 2).

Very good agreement was found for SUV/L mean, SUV/L peak, and SUV max measured in the region inferior to the portal vein and for SUV/L mean and SUL peak in the region superior to the portal vein and in SUL mean in the region of the portal vein. Absolute agreement was always higher for SUV/L mean followed by SUV/L peak and SUV/L max. SUL measurements always had better agreement compared to SUV measurements except for the area inferior to the portal vein in which SUV max demonstrated very good agreement compared to good agreement in SUL max (Table 2).

## 4. Discussion

The present study demonstrates that the area inferior to the portal vein is the most reliable location in the liver and might be the best region to be used as background for the evaluation of tumor to liver background on PET/MR.

FDG uptake can be assessed either quantitatively or qualitatively. As absolute quantitation is cumbersome and not practical in clinics, semiquantitative methods expressed as a single numeric Standardized Uptake Value (SUV) have been increasingly used for evaluating cancer patients. Standardization of SUV is crucial as this value is affected by several factors and is usually performed with a tight control of the various factors that affect SUV measurements or by using a ratio of tumor to background FDG uptake. Normal liver and blood pool (e.g., mediastinum) SUV are usually used on FDG PET/CT studies as background tissues given their high degree of repeatability. Qualitative assessment is based on visual comparison of FDG uptake in tumors with that of a single or several background tissues.

A basic requisite to any of the aforementioned methods of FDG uptake assessment is the test-retest reliability and variability of background tissues. Whenever a test-retest variability range is defined, each follow-up scan is evaluated accordingly. If changes fall in the variability range, the scan is deemed adequate and any change in tumoral FDG uptake is considered a true change and needs to be further assessed to determine its clinical significance. On the contrary, if changes are above the defined range, a search for systematic errors has to be performed and the study interpretation has to be made in the light of and with the understanding of these factors.

Several studies have investigated 18F-FDG PET/CT test-retest variability of SUV/L measurements in the liver. Boktor et al. [5] have found that test-retest variation in liver mean SUV has a mean of 0.12 ± 0.5 with a reference range of −0.9 to 1.1. They used a two-dimensional region of interest (ROI) located in the right lobe of the liver “well away from diaphragmatic motion artifacts”. Tahari et al. [3] found intrapatient variation in liver mean SUL in the range of −0.5 to 0.6. They found an average absolute test-retest difference of 0.03 ± 0.27 and ICC of 0.35–0.41, 0.37–0.38, and 0.38–0.44 superior to, at the level of, and inferior to the portal vein, respectively. Paquet et al. [6] revealed an absolute difference of 0.05 ± 0.2 and 0.05 ± 0.3 for SUL mean and SUV mean, respectively, and 0.08 ± 0.33 and 0.09 ± 0.48 for SUL max and SUV max, respectively. Only maximal SUL was statistically different between studies (*p* < 0.05). Absolute agreement (ICC) of 0.57, 0.65, 0.65, and 0.7 and CV (%) of 10.8, 12.4, 11, and 12.6 was found for mean and maximal SUL and SUV, respectively. In their study, ROI was placed in a central region in the right lobe of the liver.

To the best of our knowledge, this is the first study to investigate different SUVs and SULs measurements in different locations in the liver on 18F-FDG PET/MR. As a new modality 18F PET/MR test-retest reliability with regard to SUV/L measurements is needed. Principal factors that differ between 18F-FDG PET/CT and 18F-FDG PET/MR that might affect reliability include Dixon-based attenuation correction maps, scanning time, and MR hardware that is located in proximity to PET detectors. This has led us to determine “inherent” variation range in liver FDG uptake between studies. For instance, using the average SUV peak inferior to the portal vein, the difference in SUV between studies is −0.14; therefore if we use the 95% CI an expected range between studies is +0.27 to −0.54. This range should be taken into consideration when interpreting serial 18F-FDG PET/MR studies.

Unlike 18F-FDG PET/CT repeatability studies that showed better repeatability with SULs [7, 8], we found that SUVs showed slightly less variability and similar agreement compared to SULs measurements in the area inferior to the portal vein (8.2% and 0.84 versus 8.5% and 0.85, resp.). A very good agreement was found for SUV/L mean, SUV/L peak, and SUV max in that region. Furthermore, in general, SUV/L mean had better agreement than SUV/L peak followed by SUV/L max. This is reasonable as averaging of SUV measurements is less prone to outliner values that influence correlation. With regard to absolute variation we found that the most consistent measurements are found inferior to the portal vein followed by the area at the level of the portal vein with the least repeatable measurements seen superior to the portal vein and in the left lobe. This distribution might be explained by breathing effect on measurements that are more pronounced closer to the diaphragm and were exacerbated by difference in breathing instructions between the first and second scan. We find this interesting in light of Viner et al. [9] study results with FDG PET/CT in which the area superior to the portal vein demonstrated the highest interreader agreement regarding SUL mean, a finding that was further supported by Tahari et al. [3]. Furthermore, the area superior to the portal vein is now recommended as the preferred area to measure liver FDG uptake on PET/CT [10]. However, both studies evaluated only interreader agreement for the same time study and not repeatability for sequential studies.

Our study has several limitations. First, the number of patients is relatively small. Second, the time interval between test and retest measurements does not reflect “reality” where the time interval is much longer usually in the range of several weeks to months. On the other hand, since a low variability on liver FDG uptake has been shown with PET/CT, this might be of benefit as it evaluates PET/MR scanner performance with basically zero to minimal effect of factors that are seen in longer interval that influence reliability like changes in body habitus, changes in liver texture as a result of therapy, and so forth. Third, because the second study has focused on the liver with the addition of contrast injection, breathing instructions differed accordingly with potential effect on SUV/L measurements. It could have been better to use the same breath holding technique to evaluate the test-retest repeatability. Even so, our variability results in the area inferior to the portal vein are similar to previous reports on repeatability of PET/CT FDG uptake in the liver [1] with a CV around 10%, supporting this area as the most reliable even with different breath instructions.

In conclusion, the least variability of SUV/L measurements in the liver was demonstrated inferior to the portal vein, suggesting that this location may serve as the preferred area for background comparison on follow-up studies. Further studies are warranted to validate whether the use of other areas, especially at the portal vein area, would make any relevant differences in clinical practice.

## Figures and Tables

**Table 1:** SUV and SUL max, mean, and peak and paired difference with standard deviation measurements superior to, inferior to, and at the level of the portal vein and left lobe of the liver in first (test) and second (retest) scans.

	Superior	Inferior	Portal	Left
SUV max test	2.52 ± 0.53	2.1 ± 0.31	2.24 ± 0.44	2.47 ± 0.47
SUV max retest	2.21 ± 0.55	1.97 ± 0.38	1.99 ± 0.40	2.12 ± 0.51

Paired difference	0.31 ± 0.45	0.14 ± 0.21	0.26 ± 0.36	0.35 ± 0.48

SUV mean test	1.95 ± 0.41	1.76 ± 0.26	1.83 ± 0.33	1.92 ± 0.35
SUV mean retest	1.77 ± 0.46	1.62 ± 0.29	1.65 ± 0.32	1.7 ± 0.41

Paired difference	0.18 ± 0.26	0.14 ± 0.14	0.18 ± 0.2	0.21 ± 0.26

SUV peak test	2.19 ± 0.48	1.92 ± 0.28	1.99 ± 0.37	2.15 ± 0.4
SUV peak retest	1.9 ± 0.51	1.78 ± 0.3	1.8 ± 0.36	1.9 ± 0.42

Paired difference	0.28 ± 0.33	0.14 ± 0.16	0.19 ± 0.24	0.25 ± 0.3

SUL max test	1.79 ± 0.4	1.45 ± 0.23	1.6 ± 0.31	1.74 ± 0.32
SUL max retest	1.58 ± 0.34	1.34 ± 0.25	1.42 ± 0.27	1.5 ± 0.34

Paired difference	0.21 ± 0.33	0.10 ± 0.16	0.18 ± 0.23	0.24 ± 0.3

SUL mean test	1.4 ± 0.3	1.26 ± 0.2	1.3 ± 0.23	1.37 ± 0.25
SUL mean retest	1.26 ± 0.31	1.16 ± 0.21	1.18 ± 0.22	1.22 ± 0.29

Paired difference	0.14 ± 0.15	0.1 ± 0.1	0.19 ± 0.13	0.15 ± 0.17

SUL peak test	1.56 ± 0.35	1.37 ± 0.2	1.43 ± 0.26	1.54 ± 0.28
SUL peak retest	1.4 ± 0.32	1.26 ± 0.21	1.28 ± 0.24	1.35 ± 0. 3

Paired difference	0.17 ± 0.17	0.11 ± 0.12	0.15 ± 0.16	0.18 ± 0.21

**Table 2:** SUV and SUL: CV and ICC from duplicate measurements superior to, inferior to, and at the level of the portal vein and in the left lobe of the liver.

	SUV max		SUV peak		SUV mean		SUL max		SUL peak		SUL mean	
	ICC	CV%	ICC	CV%	ICC	CV%	ICC	CV%	ICC	CV%	ICC	CV%
Superior	0.59	17.1	0.78	14.6	0.82	11.9	0.63	16.4	0.87	11.4	0.89	10.8
Inferior	0.83	8.5	0.84	8.2	0.87	8.2	0.78	9.2	0.85	8.5	0.89	8.2
Portal	0.64	14.6	0.78	11.3	0.81	10.7	0.68	13.7	0.79	11.3	0.82	10.1
Left	0.52	18.3	0.73	13.5	0.77	12.8	0.59	16.3	0.74	13.5	0.79	12.4
